# The nature and combination of subunits used in epitope-based *Schistosoma japonicum *vaccine formulations affect their efficacy

**DOI:** 10.1186/1756-3305-3-109

**Published:** 2010-11-19

**Authors:** Xuefeng Wang, Lei Zhang, Ying Chi, Jason Hoellwarth, Sha Zhou, Xiaoyun Wen, Lei He, Feng Liu, Calvin Wu, Chuan Su

**Affiliations:** 1 Department of Pathogen Biology and Immunology, Jiangsu Key Laboratory of Pathogen Biology, Department of Pharmacology, Nanjing Medical University, Nanjing, Jiangsu 210029, PR China; 2 Central Laboratory of the Affiliated People's Hospital, Jiangsu University, Zhenjiang, Jiangsu 212002, PR China; 3 Keck School of Medicine, University of Southern California, 1975 Zonal Avenue, KAM 100B, Los Angeles, CA 90089, USA

## Abstract

**Background:**

Schistosomiasis remains a major public health problem in endemic countries and is caused by infections with any one of three primary schistosome species. Although there are no vaccines available to date, this strategy appears feasible since natural immunity develops in individuals suffering from repeated infection during a lifetime. Since vaccinations resulting in both Th1- and Th2-type responses have been shown to contribute to protective immunity, a vaccine formulation with the capacity for stimulating multiple arms of the immune response will likely be the most effective. Previously we developed partially protective, single Th- and B cell-epitope-based peptide-DNA dual vaccines (PDDV) (T3-PDDV and B3-PDDV, respectively) capable of eliciting immune responses against the *Schistosoma japonicum *22.6 kDa tegument antigen (Sj22.6) and a 62 kDa fragment of myosin (Sj62), respectively.

**Results:**

In this study, we developed PDDV cocktails containing multiple epitopes of *S. japonicum *from Sj22.6, Sj62 and Sj97 antigens by predicting cytotoxic, helper, and B-cell epitopes, and evaluated vaccine potential *in vivo*. Results showed that mice immunized with a single-epitope PDDV elicited either Tc, Th, or B cell responses, respectively, and mice immunized with either the T3- or B3- single-epitope PDDV formulation were partially protected against infection. However, mice immunized with a multicomponent (3 PDDV components) formulation elicited variable immune responses that were less immunoprotective than single-epitope PDDV formulations.

**Conclusions:**

Our data show that combining these different antigens did not result in a more effective vaccine formulation when compared to each component administered individually, and further suggest that immune interference resulting from immunizations with antigenically distinct vaccine targets may be an important consideration in the development of multicomponent vaccine preparations.

## Background

Schistosomiasis is one of the most important neglected tropical diseases (NTDs) and remains a major public health problem in endemic countries [1,2]. Although schistosomiasis can be treated with praziquantel [3], the high re-infection rate limits the overall success of chemotherapy which typically needs to be readministered multiple times during the first two decades of life [4,5]. Therefore, the development of a safe, effective vaccine could improve long-term control of schistosomiasis and improve the efficacy of chemotherapeutic interventions [6-8].

Vaccination with radiation-attenuated cercariae induced significant levels of resistance to schistosome challenge via Th1- and Th2-mediated responses in animal models of disease. However, multiple concerns regarding this method make it unsuitable for human use [9,10]. Considerable efforts have been aimed at the identification of relevant (immunoprotective) schistosome antigens resulting in the identification of potential vaccine targets [6,11,12]. The major challenge in the development of anti-schistosome vaccines is to use defined antigens to stimulate the appropriate immune response that lead to protection. Although the *S. japonicum *Sj22.6 [13], Sj62 [14], and Sj97 [15] antigens, which are all important components of schistosome adult worm antigens (SWA), have been shown to be promising vaccine candidates, other approaches have focused on eliciting specific B-cell and Th-cell responses by identifying different antigenic determinants in potential vaccine targets [16,17]. Epitope-based vaccines offer the prospect of targeted immunity resulting in safer and more effective antigen-specific immune responses [18]. Previously we developed partially protective Th-, and B-cell epitope vaccines derived from the Sj22.6 or Sj62 antigens, respectively. However, the levels of protection induced by both vaccines were limited.

In addition, type I CD8^+ ^T cells (effector CD8^+ ^T cells), which produce INF-γ, have been proposed to play an immunoregulatory role during schistosomiasis by dampening immunopathologic type 2 responses [19,20]. Studies of the Sm28GST vaccine suggest that both CD4^+ ^and CD8^+ ^T cells might contribute to protection. Activation of Sm28GST-specific CD8^+ ^T cells produced high levels of gamma interferon (IFN-γ) involved in protective immune responses, which suggest that CD8^+ ^T-cell response induced by an antigen from the extracellular parasite *S. mansoni *may protect the mice from infection [21,22].

Currently, there are numerous efforts focused on optimizing schistosome vaccines (and vaccines against other infectious agents) using multiple-antigen or multiple-epitope design [23-26]. One strategy consists of designing subunit constructs containing defined B- and T-cell stimulatory epitopes obtained by genetic engineering or by chemical synthesis [27,28]. In some experimental models, anti-repetitive peptide responses have been able to confer immune protection against infection [29,30].

In this report we used the full-length *S. japonicum *vaccine candidates Sj22.6, Sj62 and Sj97 to generate eight distinct computer-based eptiopes identified by their potential for eliciting Tc-, Th-, or B-cell responses, respectively, using computer-based epitope-predicting software. All eight epitopes (named C1, C2, C3, B1, B2, B3, T2 and T3) were synthesized and encapsulated with the corresponding recombinant eukaryotic plasmid DNA encoding the corresponding epitope, respectively, to construct a peptide-DNA dual vaccine (PDDV) that has an antigenic peptide "shell" and a plasmid "nuclei". These pseudotype virus-like particles have revealed tremendous potential as novel delivery systems to enhance cell-specific gene delivery [31,32] and efficiently stimulate the host immune reponses [33,34]. We examined whether multicomponent PDDVs consisting of Tc (C)-, Th (T)- and B-cell (B) epitopes were more effective formulations against *S. japonicum *challenge than T- or B-cell single-epitope PDDVs. Our data showed that vaccination of mice with single-epitope-PDDV elicited corresponding immune responses - *i.e*., cytotoxicity, proliferation, or antibody production, respectively - and that vaccination with T3- or B3-PDDV induced partial protection. However, vaccination of mice with multicomponent PDDV formulations comprised of multiple epitopes produced variable immune responses that failed to induce better protection than T3- or B3- single-epitope PDDVs.

## Results

### Epitopes and their encoding DNA sequences

As shown in Table 1, eight candidate epitopes (C1, C2, C3, T2, T3, B1, B2, and B3), plus an 18K tail respectively, were chosen based on their predicted antigenicity scores for the further examination of various anti-*S. japonicum *immune responses. The DNA sequences used to encode the respective *S. japonicum *epitopes were designed based on the published *S. japonicum *DNA sequences for Sj22.6 [13], Sj62 [14] and Sj97 [15].

**Table 1:** Design of peptides used in the construction of the PDDVs.

Code	Amino acid sequence of 18 Lys and the 18 Lys fused epitopes synthesized for PDDV formulations	Oligonucleotide sequences used for plasmid construction	Source	Epitope type
18K	KKKKKKKKKKKKKKKKKK	None	None	None
C1	KKKKKKKKKKKKKKKKKKNLMKENKNL	5'-tcgacatgaatcttatgaaagaaaataagaatttag-3'	Sj 97 (555-563)	CTL
		5'-aattctaaattcttattttctttcataagattcatg-3'		
C2	KKKKKKKKKKKKKKKKKKVRAVANDLK	5'-tcgacatggtaagagcggtggcaaatgacttaaaag-3'	Sj 22.6 (134-142)	CTL
		5'-aattcttttaagtcatttgccaccgctcttaccatg-3'		
C3	KKKKKKKKKKKKKKKKKKATRLNNEVL	5'-tcgacatggcaactagattgaataatgaagttttgg-3'	Sj 97 (672-680)	CTL
		5'-aattccaaaacttcattattcaatctagttgccatg-3'		
T2	KKKKKKKKKKKKKKKKKKITELEDVAERERLKA	5'-tcgacatgatcactgaacttgaagatgttgcagagagagaacgattaaaagcgg-3'	Sj 97 (313-327)	Th-cell
		5'-aattccgcttttaatcgttctctctctgcaacatcttcaagttcagtgatcatg-3'		
T3	KKKKKKKKKKKKKKKKKKAKQYNICCKFKELLD	5'-tcgacatggctaagcaatataacatatgttgtaaatttaaagaacttctcgatg-3'	Sj22.6 (111-125)	Th-cell
		5'-aattcatcgagaagttctttaaatttacaacatatgttatattgcttagccatg-3'		
B1	KKKKKKKKKKKKKKKKKKEQRLRERDEELESLRKSTTRTI	5'-tcgacatggaacagagacttagagaaagagatgaagaattagaaagtctaagaaagagtacaactagaacaatattg-3'	Sj 97 (489-510)	B-cell
		5'-aattcaatattgttctagttgtcatctttcttagactttctaattcttcatctctttctctaagtctctgttccatg-3'		
B2	KKKKKKKKKKKKKKKKKKWEVRREKEELKKDKEGKVSTL	5'-tcgacatgtgggaagtccgtcgtgaaaaggaagaattaaagaaagacaaggaaggcaaagtatccacacttg-3'	Sj 22.6 (77-97)	B-cell
		5'-aattcaagtgtggatactttgccttccttgtctttctttaattcttccttttcacgacggacttcccacatg-3'		
B3	KKKKKKKKKKKKKKKKKKRQEEEMKKAAEELAKLKEEFEK	5'-tcgacatgcgtcaggaagaagaaatgaagaaagcagccgaagaattagctaaactaaaagaagaatttgaaaaag-3'	Sj 62 (167-188)	B-cell
		5'-aattctttttcaaattcttcttttagtttagctaattcttcggctgctttcttcatttcttcttcctgacgcatg-3'		

### C2- and C3-PDDV induced the cytotoxic effect and elicited antibody and IFN-γ production in C57BL/6 mice

**Figure 1:**
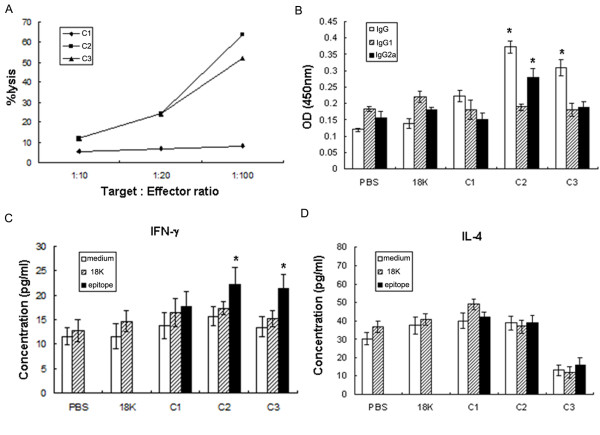
**CTL-PDDVs induced the cytotoxicity effect and produced antibody**. (A) Effect of C-PDDV vaccination on cytotoxicity. Seven days after the last C-PDDV, 18K-PDDV, or PBS immunization epitope-specific cytotoxic activity was measured by incubating murine spleen effector cells or p815 target cells with either C1-, C2-, or C3-18K fusion peptides, 18K control peptide, or medium only, then mixed cells at E:T rations ranging from 1:10 to 1:100. The CTL activity of the cells was tested using Na_2_[^51^Cr]O_4 _assay. Data are expressed as the mean ± SD (n = 6 per group) of 18 mice from three independent experiments performed in triplicate wells. (B) Serum antibody subtype profile following C-PDDV vaccination. Whole IgG, IgG1, and IgG2a responses to SWA (0.1 mg/ml) following vaccination with C-PDDV formulations, or controls were measured by ELISA. Data are expressed as the mean ± SD (n = 6 per group) of 18 mice from three independent experiments performed in triplicate wells. * *P *< 0.05 and ** *P *< 0.01, compared to the 18K-PDDV and PBS groups. (C-D) Cytokine profile analysis following C-PDDV vaccination. IFN-γ (C) and IL-4 (D) production of splenocytes harvested from every vaccination group were determined by culturing in triplicate. In 96-well plates, 10^6^cells/well were cultured for 48 h in 200 μl of media in the presence of C1-, C2-, C3-18K fusion peptide (10 μg/ml), 18K (10 μg/ml), or media alone. Supernatants were collected after 48 h of culture for cytokine detection. Bars show the mean ± SD (n = 6 per group) of 18 mice from three independent experiments performed in triplicate wells.

Cytotoxic responses to epitopes predicted to elicit Tc responses were measured by immunizing mice with either C1-PDDV, C2-PDDV, or C3-PDDV. Control mice were immunized with either 18K-PDDV or PBS. Splenocytes harvested from immunized mice demonstrated that C2- and C3-PDDV immunizations elicited the strongest cytotoxic responses (Figure 1A). A significant IgG response was elicited by C2- and C3-PDDV (but not C1-PDDV or controls), and C2-PDDV-immunized mice also developed a significantly elevated IgG2a response. However IgG1 responses were not observed in mice immunized with any of the CTL-PDDV formulations (Figure 1B). Analysis of IFN-γ (Figure 1C) and IL-4 (Figure 1D) production of cultured splenocytes from PBS, control 18K PDDV, C1-, C2-, or C3-PDDV vaccinated mice did not reveal significant increases in IL-4 production. However, following restimulation with the respective C2- or C3-18K fusion peptide, a statistically significant increase in IFN-γ production was observed compared to controls restimulated with medium or 18K *in vitro*. These results indicate that C2- and C3-PDDV vaccination induced cytotoxic responses associated with IFN-γ production and IgG and IgG2a production.

### T3-PDDV induced both cellular and humoral immune responses in C57BL/6 mice

**Figure 2:**
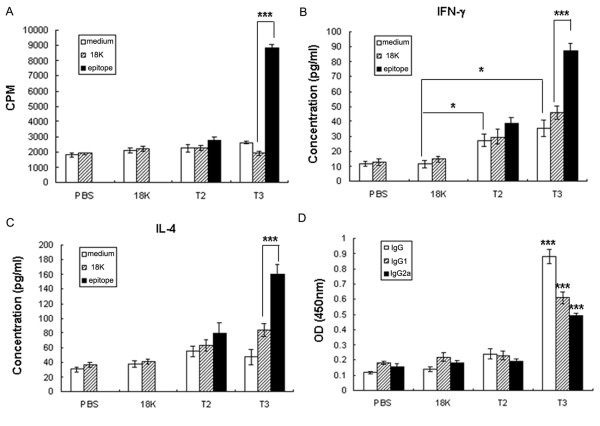
**T3-PDDV induced both cellular and humoral immune responses**. (A) T-PDDV induced cellular immunity. Seven days after the last immunization with T-PDDVs, 18K-PDDV, or PBS, splenocytes were harvested and antigen-specific proliferation was measured. Splenocytes (2 × 10^5^/well) from each mouse were incubated in triplicate for three days in 200 μl in 96-well plates in the presence of either the T2-18K, T3-18K, or 18K peptides (10 μg/ml), or media alone. To each well 0.5 μCi [^3^H] thymidine was added 16 h before the end of the incubation period. Data are expressed as the mean ± SD (n = 6 per group) of 18 mice from three independent experiments performed in triplicate wells. *** *P *< 0.001. (B-C) Cytokine production following T2- or T3-PDDV vaccinations. IFN-γ (B) and IL-4 (C) production was measured in splenocytes harvested and cultured from the respective vaccination groups for 48 h. Bars show the mean ± SD (n = 6 per group) of 18 mice from three independent experiments performed in triplicate wells. * *P *< 0.05; *** *P *< 0.001. (D) IgG, IgG1, and IgG2a responses in immunized mice. Antibody responses to SWA (0.1 mg/ml) were determined by ELISA. *** *P *< 0.001, compared with 18K-PDDV and PBS groups. Data are expressed as the mean ± SD (n = 6 per group) of 18 mice from three independent experiments performed in triplicate wells.

To examine the immune responses induced by the T-PDDVs vaccination, both cellular and humoral immune responses were analyzed after the final vaccination. Only splenocytes harvested from T3-PDDV-vaccinated mice proliferated in response to T3-18K fusion peptide stimulation in comparison to 18K-peptide or medium stimulated controls (Figure 2A). Both T2- and T3-PDDV-immunized mice produced significant levels of IFN-γ in the absence of *in vitro *restimulation with T2- or T3-18K fusion peptide, suggesting that T2 and T3-PDDV vaccination induced specific Th1-type cellular immune responses *in vivo*. However, *in vitro *restimulation of splenocytes harvested from T3-PDDV-vaccinated mice with T3-18K fusion peptide resulted in a significant increase of IFN-γ production (Figure 2B). T3-PDDV-immunized mice also developed a significant IL-4 response following *in vitro *restimulation with the T3-18K fusion peptide, suggesting that T3-PDDV could also induce the production of a Th2-associated cytokine (Figure 2C). Compared to both control groups, only T3-PDDV-immunized mice developed significant antigen-specific IgG, IgG1, and IgG2a responses (Figure 2D). These results suggest that immunization with T3-PDDV induced both Th1/Th2 and antibody responses in C57BL/6 mice.

### B3-PDDV induced the highest antibody response in C57BL/6 mice

**Figure 3:**
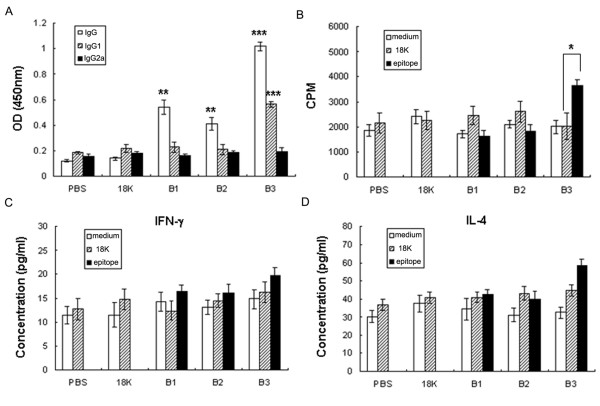
**B3-PDDV induced the highest antibody response**. (A) Analysis of B-PDDV-induced antibody responses. Seven days after the last immunization with B-PDDVs, 18K-PDDV, or PBS, mouse whole IgG, IgG1, and IgG2a antibody responses to SWA (0.1 mg/ml) were analyzed by ELISA. Data are expressed as the mean ± SD (n = 6 per group) of 18 mice from three independent experiments performed in triplicate wells. ** *P *< 0.01 and *** *P *< 0.001, compared to the 18K-PDDV and PBS groups. (B) Splenocyte proliferation assay. Splenocytes (2 × 10^5^/well) from each mouse were incubated in triplicate wells for three days in 200 μl of media in 96-well plates in the presence of B1-, B2-, B3-18K fusion peptide, or 18K (10 μg/ml) peptides (or media alone). Proliferation was determined by measuring [^3^H] thymidine incorporation for the last 16 h of the experiment. Data are expressed as the mean ± SD (n = 6 per group) of 18 mice from three independent experiments. * *P *< 0.05. (C-D) Cytokine production analysis. Supernatants were collected after 48 h of culture and examined for IFN-γ (C) or IL-4 (D). Bars show the mean ± SD (n = 6 per group) of 18 mice from three independent experiments performed in triplicate wells.

To investigate the immune responses induced following immunization with the B-PDDV epitopes, mice were immunized with either B1-, B2- or B3-PDDV or the 18K-PDDV or PBS, respectively. Compared to the both 18K-PDDV and PBS control groups, all three B-PDDV groups developed significantly increased IgG responses. However, only B3-PDDV-immunized mice developed a significant IgG1 response (Figure 3A). Furthermore, only splenocytes harvested from B3-PDDV-immunized mice proliferated significantly in response to *in vitro *stimulation with the B3-18K fusion peptide (Figure 3B). Cytokine production analysis of supernatants showed that none of the B-PDDVs elicited significant increases in IFN-γ or IL-4 production (Figure 3C and 3D) suggesting that these epitopes mainly elicited significant changes in antibody production.

### Cytokine and antibody responses in mice vaccinated with CTL-, T- and B-PDDV cocktails

**Figure 4:**
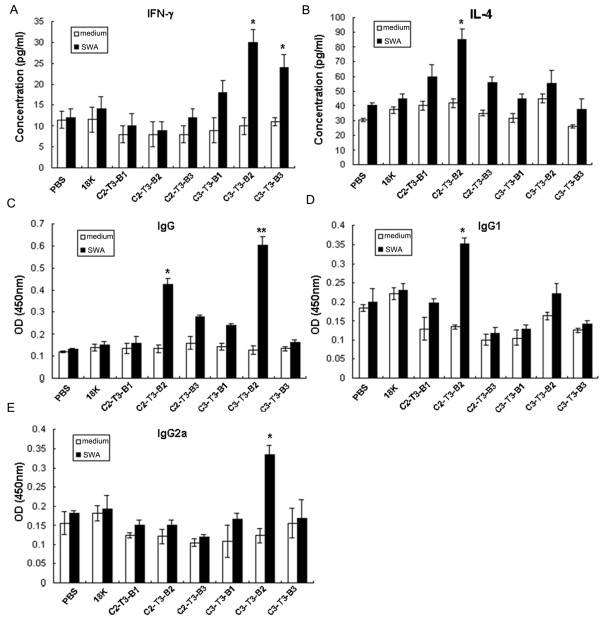
**Cytokines and antibodies responses in mice vaccinated with CTL-, T-, and B-PDDV cocktails**. (A-B) Cytokine responses following vaccination with C-, T- and B-PDDV multicomponent formulations. Seven days after the last immunization with either multicomponent PDDV formulations, 18K-PDDV, or PBS, splenocyte production of IFN-γ (A) and IL-4 (B) from each vaccine group was assessed. Splenocytes from each mouse were cultured (10^6^/well) in triplicate for 48 h in the presence or absence of SWA (50 μg/ml). Supernatants were collected after 48 h and IFN-γ and IL-4 production was assessed. The data are expressed as the mean ± SD (n = 6 per group) of 12 mice from two independent experiments performed in triplicate wells. (C-E) Antibody responses following multicomponent vaccinations. IgG (C), IgG1 (D), and IgG2a (E) responses to SWA in immunized mice were determined by ELISA. Data are expressed as the mean ± SD (n = 6 per group) of 12 mice from two independent experiments performed in triplicate wells. *P < 0.05 and ***P < 0.001, compared to the 18K-PDDV and PBS groups.

Previous studies have shown that CD8^+ ^cells play a regulatory role in schistosomiasis through the regulation of cytokines, affecting the immune response and immune pathology [19]. To test whether the CTL-PDDV-induced CD8^+ ^T cells were able to improve the immune response induced by T- and B-PDDV, and to further investigate whether the multicomponent PDDV formulations could result in better immune effection, mice were immunized with multicomponent PDDV preparations containing three different types of single PDDVs at a 1:1:1 ratio, and the levels of cytokines in culture supernatants and antibodies in sera of vaccinated mice were measured. C1 and T2-PDDV were not selected for further study because of their poor immune responses in above studies, only C2, C3, T3, and B-PDDVs were used. As shown in Figure 4A, mice vaccinated with equal concentrations of different PDDV formulations revealed that IFN-γ production was statistically elevated in the C3-T3-B2 and C3-T3-B3-vaccinated groups following restimulation *in vitro *with SWA containing Sj22.6, Sj62, and Sj97 proteins (Figure 4A). In contrast, IL-4 levels were statistically elevated in mice vaccinated with C2-T3-B2 following SWA *in vitro *restimulation (Figure 4B). Compared to the both 18K-PDDV and PBS control groups, only C2-T3-B2 and C3-T3-B2 groups induced significantly increased IgG responses (Figure 4C). However, only C2-T3-B2-immunized mice developed a significant IgG1 response (Figure 4D), with IgG2a antibody only elicited in the C3-T3-B2 group (Figure 4E). These data suggest that different epitope combinations favore different cytokine and antibody subclass production profiles, highlighting the importance of selecting the appropriate antigen combination for the induction of the immune responses most likely to elicit protective immunity.

### PDDV cocktails did not improve the resistance to *S. japonicum *infection

The protective effect conferred by the different PDDV or PDDV multicomponent formulations was determined by quantifying worm and egg (liver) burdens in the respective vaccination/infection groups (Table 2). Consistent with results described previously by our group [13,14], T3- and B3-PDDV vaccinations induced significant reductions in worm and egg burdens in this experiment. Vaccination with T3-PDDV resulted in a 35.80% worm burden reduction and a 51.60% egg (liver) reduction compared to 18K-PDDV-vaccinated controls. Similarly, vaccination with B3-PDDV resulted in a 19.10% worm reduction and a 29.20% egg (liver) reduction compared to the 18K-PDDV-vaccinated controls. Meanwhile, only mice immunized with C3-T3-B2 were partially protected (more than 10% protection in both worm and egg reductions compared to controls). However, the data suggests that multicomponent formulations (using the epitopes described) failed to improve the resistance to *S. japonicum *infection compared to single epitope (T3 or B3) PDDV vaccination.

**Table 2:** Worm and egg burdens following vaccination.

Vaccination Regimen	Mean worm count ± SD	Mean liver egg count ± SD	Worm reduction rate (%)		Liver egg reduction rate(%)	
			
			Compared to the PBS group	Compared to the 18K group	Compared to the PBS group	Compared to the 18K group
C2	31.33 ± 6.66	20900.9 ± 1899.84	-31.3	-34.5	10.08	3.7
C3	24.50 ± 4.23	18558.54 ± 2120.31	-2.5	-5.2	20.16*	14.5
T3	14.50 ± 8.39	10270.27 ± 4357.82	39.33**	35.80**	55.81**	51.60**
B1	23.71 ± 7.18	20308.88 ± 3154.43	0.78	0	12.62	6.5
B2	20.40 ± 6.47	21513.51 ± 4128.92	14.64	12.4	7.44	0.9
B3	18.86 ± 3.35	15366.78 ± 2371.41	22.70*	19.10*	34.81**	29.20*
C2-T3-B1	24.80 ± 6.98	19243.24 ± 2172.86	-3.8	-6.4	17.21	11.4
C2-T3-B2	18.75 ± 1.73	20000.0 ± 2167.85	21.55*	19.5*	13.95	7.9
C2-T3-B3	22.57 ± 7.14	22162.16 ± 4496.31	5.56	3.1	-2.1	4.7
C3-T3-B1	25.75 ± 6.70	18648.65 ± 4105.15	-7.7	-10.5	19.77*	14.1
C3-T3-B2	20.00 ± 6.40	12540.54 ± 4658.24	16.32	14.2	46.05**	42.2**
C3-T3-B3	21.33 ± 2.83	15855.84 ± 4944.18	10.74	8.4	31.78*	27.0*
18K	23.33 ± 6.41	21711.70 ± 3937.61	/	/	/	/
PBS	23.88 ± 7.91	23243.24 ± 4784.20	/	/	/	/

The mean worm/liver egg burdens were calculated using results from sixteen mice from two independent experiments (n = 8). Percent protection in the two independent experiments was calculated by comparing their results with the results obtained from the 18K-PDDV and PBS groups. Results are expressed as means ± standard errors. *P < 0.05; **P < 0.01, compared to the worm/liver egg burdens of the 18K-PDDV and PBS vaccination groups.

### Histopathology of egg granulomas in mice livers

**Figure 5:**
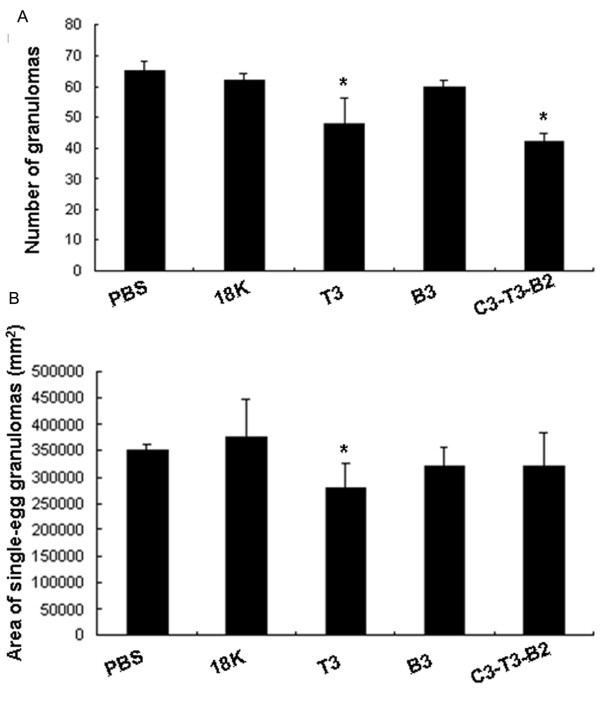
**Histopathology of egg granulomas in the livers**. (A) Six weeks after challenge, mice were sacrificed. After portal perfusion, livers were dissected and stained with H&E for microscopic examination. The number of granulomas in the liver of each mouse was counted in 10 random fields and the data are expressed as the mean ± SD of 16 mice (8 mice/group) from two independent experiments. (B) The size of nonconfluent granulomas formed around a single egg was assessed using a video micrometer. The data are expressed as the mean ± SD of 16 mice (8 mice/group) from two independent experiments. *P < 0.05, compared to the 18K-PDDV and PBS groups.

Based on the protection results described above, we selected the vaccination groups with highest egg reductions for mice liver histologic examination. Compared to both control groups, the average number of egg granulomas in 10 random fields after the challenge infection was significantly decreased in the livers of mice immunized mice with T3-PDDV or with the C3-T3-B2 PDDV preparation (Figure 5A). In addition, the mean-area of non-confluent granulomas in these livers was statistically smaller in T3-PDDV-vaccinated mice. These data suggest that immunization with the T3-PDDV or C3-T3-B2 PDDV reduced the number and/or size of egg granulomas (Figure 5B).

## Discussion

The development of vaccines for complex parasites such as schistosomes is a great challenge. Vaccination with radiation-attenuated cercariae induces significant levels of resistance to schistosome challenge and suggests the fact that vaccination induced both B and T cell responses are critical, and combination of different antigens may be an efficient strategy to improve the immunoprotection against schistosome infection. In this study, different schistosome vaccine candidates (alone or mixed) that stimulated either B or T cell immunity were tested in a mouse model of disease. Results showed that combining different antigens in fact did not result in a more effective vaccine formulation when compared to each component administered individually, suggesting that mixed antigens may not be necessary for protection against schistosome infections and that immune interference resulting from the inoculation with multiple antigens is one mechanism responsible for the unexpected lack of increased protection.

Many years devoted to studying the interactions between schistosome infection and the resulting immune responses by hosts has led to the identification of mechanisms believed to play a critical role in protective immunity. Studies have shown that protection elicited by vaccination is not dependent on one immune mechanism but is multifactorial, involving both cellular and humoral elements and can be affected by the host's genetic background and the vaccine regimen [35,36]. Therefore, to improve anti-schistosome immunity and protection against infection conferred by respective vaccine formulations, various strategies including multiple antigenic peptides (MAPs), *i.e*. sequential arrangement of epitopes into a single polypeptide and multicomponent formulations, have been tested [17,23,24]. These vaccination modalities have been tested in animal models and some formulations have proved successful [37,38], suggesting that multicomponent formulations can be used to develop effective anti-schistosome vaccines. Recently, we demonstrated that vaccination with PDDV based on a T cell-epitope (P5, in this study named T3) derived from the Sj22.6 tegument antigen or a B cell-epitope (B3) derived from the Sj62 myosin sequence all induced partial protection against *S. japonicum *challenge in mice [13,14]. In this study we predicted and selected six additional peptide epitopes derived from full-length Sj22.6, Sj62, and Sj97 antigens shown to have the potential of eliciting protective immunity in various other studies [6,13,14,39-42]. The experiments were aimed at improving the immunoreactivity and protective efficacy of the peptide-based approach by comparing immune responses following vaccinations with either single or multicomponent formulations in mice subsequently infected with *S. japonicum*. Results from mice vaccinated with Tc-, Th-, or B-PDDV single-epitope formulations showed that not every single-epitope PDDV elicits corresponding cytotoxic, T helper or antibody responses. This finding suggests that at present, computer assisted epitope prediction is not a very effective way of generating epitopes and experimental validation must remain an early step in any vaccine study.

In contrast to the immune response induced by single PDDVs, cytokine release and antibody production induced by multicomponent PDDV formulations showed varied immune response profiles and variability in protective immunity conferred following vaccination with multicomponent formulations was not always more effective than that observed in mice immunized with single component PDDV preparations. For example, among multicomponent PDDV formulations, only C2-T3-B2, C3-T3-B2, and C3-T3-B3-immunized mice induced partial protection in reducing worm or egg burdens, but all multicomponent PDDV formulations containing T3- or B3-PDDV were not more effective in reducing worm and egg burdens nor did multicomponent formulations elicit significant changes in cytokine or antibody production, when compared to mice immunized with single formulations of T3- or B3-PDDV. These data suggest that T3- or B3-PDDV appeared to elicit a protective response against the parasite, but other epitopes or multicomponent formulations failed to induce an adequate immune response to improve the protection. Furthermore, these results also indicate that combinations of different types of antigenic epitopes may result in immune interference resulting in the development of inefficient immune responses incapable of conferring protective immunity [43,44]. Others have shown that multicomponent vaccines can be more immunogenic and produce significant anti-parasite activity [45-47]. Similarly, DNA vaccines containing multiple antigens also contributed to improved protective responses against *Schistosoma*. For example, using a DNA vaccine encoding Sj62, Sj28, Sj23, and Sj14-3-3 induced significant Th1-type cellular responses and conferred partial protection against *S. japonicum *infection [48]. However, others have also reported different results in relation to multicomponent formulations *e.g*., a multicomponent vaccine based on antigenic epitopes derived from SmTPI, Sm28, Sm97, Sm23, and Smcalpain of *S. mansoni *did not elicit a response capable of parasite killing *in vivo *[49]. Several studies have addressed the properties of an epitope-specific regulatory system [50,51] that selectively controls immune response (such as IgG antibody production) to the individual determinants on a complex antigen. These regulatory responses are commonly believed to regulate the amount, affinity, and isotype composition of antibody responses to individual epitopes on complex antigens by interference [51].

In this study, even though we failed to demonstrate improved protective efficacy using a multiple component-based vaccine strategy, we still derived insights regarding effective design of *S. japonicum *vaccines in two ways. First, predicting epitopes with software alone is not sufficient. A combination of epitope prediction and experimental screening is needed. Second, multiple epitope vaccines capable of inducing protective antibody and cell mediated immune responses against different schistosomal developmental stages theoretically may be more effective, however, the multivalent vaccine construct must be well designed to not suffer the effects of epitope interference.

## Conclusion

In conclusion, we have developed single PDDVs and multicomponent PDDV anti-*S. japonicum *formulations. Our experiments demonstrate that mice immunized with single PDDV formulations were partially protected against *S. japonicum *infection and that mice vaccinated with multicomponent formulations were not necessarily better protected against *S. japonicum *challenge. These data suggest that immune interference may account for the inefficiency of the multicomponent formulations and that care must be taken in the selection of epitopes identified for vaccine preparations containing multiple epitopes.

## Methods

### Animal studies and antigen preparation

Six-week-old C57BL/6 female mice were provided by the Center of Experimental Animals (Nanjing University, Nanjing, PR China). *Oncomelania hupensis *harboring *S. japonicum *cercariae (Chinese mainland snail strain) were purchased from the Jiangsu Institute of Parasitic Diseases (Wuxi, PR China). All animal experiments were performed in accordance with the Chinese laws for animal protection and with permission from the Institutional Review Board. Soluble schistosome worm antigen (SWA) was prepared as previously described [52].

### Identification of antigenic epitopes

Selection of B-cell epitopes was based on predictions made by the Immune Epitope Database and Analysis Resource (IEDB; http://epitope2.immuneepitope.org/home.do) [53] and ProtScale http://www.expasy.org/cgi-bin/protscale.pl[14,54]. All putative T-cell epitopes were predicted using GUATIF, TEPITOPE and ANTHIWHIN software [13,55]. Briefly, the amino acid sequences for Sj22.6 (GenBank Accession No: AAC67308), Sj62 (GenBank Accession No: AAC82332), and Sj97 (GenBank Accession No: Q05870) were analyzed by software designed to predict the epitopes and candidate peptides most likely to elicit Tc-, Th-, or B-cell responses selected based on their respective prediction scores. The 8 selected epitopes containing an 18 Lys (18K) N-terminal tail (epitope-18K fusion peptides) were synthesized and an 18K control peptide was also synthesized and purified (Invitrogen, Shanghai, PR China). The purity of the peptides determined by mass spectrometry was >99%. The DNA sequences encoding each of the 8 identified epitopes were synthesized and purified based on the published *S. japonicum *DNA sequences, respectively, for Sj22.6 (GenBank Accession No: AF030404), Sj62 (GenBank Accession No: No. AF039187), and Sj97 (GenBank Accession No: EU488866) (Invitrogen). *Sal*I and *EcoR*I restriction sites were included in the primer sequences for cloning purposes.

### Preparation of PDDVs

PDDVs encoding the eight antigenic epitopes were prepared and confirmed as described previously [13,14]. The diagram of PDDV preparation was shown in Additional file 1. Briefly, the recombinant expression plasmid pUMVC1-mGM-CSF (a gift from Professor Yuzhang Wu, Institute of Immunology of the Third Military Medical University, Chongqing, PR China) was 4423 base pairs (bp) long and contained the cytomegalovirus (CMV) promoter and the mouse GM-CSF gene. The two complementary single stranded oligonucleotides encoding each respective epitope were annealed and inserted into the pUMVC1-mGM-CSF vector and the resulting plasmids transformed into *Escherichia coli *DH5α grown in Luria Bertani (LB) broth. The recombinant plasmid DNAs were purified using the QIAGEN Endofree Plasmid Maxi Kit (QIAGEN, Hilden, Germany). Plasmid preparations were resuspended in ddH_2_O to a final concentration of 1.5-2.0 mg/ml. Agarose gel electrophoresis confirmed that the plasmid preparations were not contaminated with bacterial genomic DNA or RNA. Preparation of the PDDVs was performed by titrating peptide into a solution of DNA containing 10 mM HEPES and 150 mM NaCl as described previously [13,14]. The cationic poly-lysine in 18K control peptide or epitope-18K fusion peptide was bound to the anionic plasmid DNA (containing the sequence of corresponding epitope) through electrostatic interactions and the peptide-DNA complex (PDDV) was condensed into nanometric pseudotype virus-like particles. Each PDDV was adjusted with phosphate buffered saline (PBS, pH 7.4) to 100 μl containing of 28 μg of peptide and 10 μg of plasmid. The PDDV containing either the control 18K or the Tc-, Th-, or B-cell epitope-18K fusion peptide were designated as 18K-PDDV, C-PDDV, T-PDDV or B-PDDV, respectively. The integrity of the PDDVs was confirmed using the DNA retardation assay, DNase I digestion assay and transmission electron microscopy as described previously [13,14].

### Immunization and challenge infection

For immune response characterization, three independent experiments were carried out. In each experiment, C57BL/6 mice (6 mice per group) were injected subcutaneously (s.c.) in the back with 100 μl of PBS (control 1), 18K-PDDV (control 2), C-, T-, or B-PDDV per mouse, respectively. The immunization was repeated three times at 14-day intervals. One week after the final vaccination, mice were sacrificed for the characterization of cellular and humoral immune response.

For vaccination/challenge trial, two independent experiments were carried out. In each experiment, C57BL/6 mice were divided into fourteen groups consisting of 14 mice per group. Each mouse was injected subcutaneously (s.c.) in the back with 100 μl of PBS (control 1), 18K-PDDV (control 2), C-, T-, B-PDDV, or multicomponent PDDV preparations, respectively. The multicomponent PDDV preparations were prepared by mixing the single PDDVs at a 1:1:1 ratio (33.3 μl each of C, T- and B-PDDVs, respectively) consisting of 28 μg of peptides and 10 μg of plasmids in total. The immunization was repeated three times at 14-day intervals. One week after the final vaccination, six mice from each group were sacrificed for the cytokine and antibody detection. Two weeks after the final vaccination, the remaining eight mice from each group were challenged percutaneously with 40 ± 1 *S. japonicum *cercariae. Six weeks later the mice were sacrificed and perfused to determine worm burdens and the liver egg burdens. Reductions in worms/liver egg burdens are expressed as a percentage of the burden recorded in the control groups.

### Cytotoxicity assay

Cytotoxicity was determined by a 4 h ^5l^Cr release assay as described previously [56]. Briefly, spleen cells were harvested from PBS, C1-, C2-, C3-PDDV or 18K-PDDV immunized mice and resuspended at a concentration of 1 × 10^6^/ml in complete 1640 medium (containing 10% FCS, 100 U/ml penicillin, 100 μg/ml streptomycin) containing 10 μg/ml C1-, C2-, C3-18K fusion peptide, 18K control peptide or medium only. After a five-day incubation at 37°C, the cells were washed and used as effector cells and 5 × 10^6 ^p815 (H-2^d^) cells were labeled with 200 μCi Na^2^[^51^Cr]O_4 _for 1 h. After thorough washing, labeled p815 cells were used as target cells and pulsed with 10 μg/ml C1-, C2-, C3-18K fusion peptide, 18K control peptide or medium only for 2 h at 37°C, washed and resuspended in complete RPMI 1640 at a concentration of 1 × 10^5^/ml. Effector cells were titrated by serial dilution in U-bottom 96-well plates at Effector/Target ratios of 100:1, 20:1, 10:1. 1 × 10^4 ^target cells were added, centrifuged for 30 s at 100 × g and the Effector-Target-cell mix incubated at 37°C, 5% CO_2_, 90% humidity for 4 h. After centrifugation at 250 × g for 10 min, 100 μl/well supernatant from respective wells was removed and CPMs were measured with a gamma counter (Beckman, Fullerton, USA). The percent specific lysis was determined using the following equation: 100 × [(experimental release-spontaneous release)/(maximum release-spontaneous release)], with spontaneous release measured as the counts obtained from target cells incubated in medium alone, and maximum release determined by the counts obtained from target cells exposed to 1% Triton X-100.

### Enzyme-linked immunosorbent assay (ELISA)

Serum samples were collected seven days after the last immunization. Standard ELISAs were performed using SWA as the antigen source [39,52]. Antibody detection in the sera of immunized mice was performed as previously described [13,14]. IFN-γ and IL-4 levels in the supernatants of splenocytes stimulated by antigens from PBS, 18K-PDDV, C-, T-, B-PDDV, or multicomponent PDDV immunized mice were measured by ELISA using the eBioscience ELISA Ready-set-Go kit (eBioscience, San Diego, USA), according to the manufacturer's instructions.

### Splenocyte proliferation assay

[^3^H] thymidine (^3^H-TdR) incorporation was used to measure splenocyte proliferation. Seven days after the last immunization, six mice from each group were sacrificed and splenocytes harvested. In 96-well plates, 2 × 10^5 ^cells per well were incubated for 72 h in 200 μl of complete media in the presence of the respective epitope-18k fusion peptides (10 μg/ml) or the 18K control (10 μg/ml). After 56 h in culture, [^3^H] thymidine (0.5 μCi) (Amersham, Burkinghamshire, UK) was added to each well. At the end of the incubation period, the cells were harvested on filters and the incorporated [^3^H] thymidine counted.

### Histopathological examination

After portal perfusion, livers were dissected and immediately fixed in 10% buffered formalin for morphometric analysis. Liver sections were embedded in paraffin and stained with hematoxylin and eosin (H&E) for microscopic examination of granulomas at 4× (Olympus, Tokyo, Japan) following sectioning. The number of granulomas in the liver of each mouse was counted in 10 random fields. The size of nonconfluent granulomas formed around single eggs was assessed using a video micrometer (Olympus, Tokyo, Japan) in accordance with the manufacturer's instructions.

### Statistical analysis

The statistical analysis was performed using SPSS version 10.1 (Statistical Package for Social Sciences, Chicago, IL statistical software). Statistical significance was determined by Student's *t*-test with *P *< 0.05 considered statistically significant.

## List of abbreviations

PDDV: peptide-DNA dual vaccine; s.c: subcutaneously; CTL: cytotoxic T lymphocyte *S.japonicum*: *Schistosome japonicum*; SWA: soluble schistosome worm antigen.

## Competing interests

The authors declare that they have no financial, professional or personal competing interests related to this article. The funding agencies played no role in the design or implementation of the study, analysis or interpretation of the data, or the preparation and submission of the manuscript.

## Authors' contributions

XFW designed and performed the study, managed, analyzed, and interpreted the data, and prepared the manuscript; LZ designed the study, facilitated and assisted the study implementation; YC, JH, SZ and XYW assisted in the design and study implementation and/or revised the manuscript; LH, FL and CW assisted in the design of the study, data analysis; CS designed the study, supervised the study implementation and revised the manuscript. All authors read and approved the final manuscript.

## Supplementary Material

Additional file 1 **Schematic diagram of forming a PDDV**. A cationic antigenic peptide containing 18 lysines (18K) and the antigenic epitope was designed and synthesized. An anionic plasmid containing the DNA sequence of the corresponding antigenic epitope sequence and mouse GM-CSF was constructed. The cationic peptides and corresponding anionic plasmids form virus-like particles through electrostatic interactions at an appropriate charge ratio of peptide and DNA. Click here for file
