# Supplementary figures and images for: The nature and combination of subunits used in epitope-based Schistosoma japonicum vaccine formulations affect their efficacy
Source: Parasit Vectors. 2010 Nov 19;3:109. doi: 10.1186/1756-3305-3-109 (PMC3136145; doi:10.1186/1756-3305-3-109)

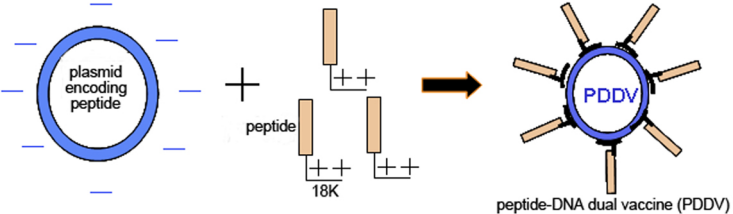

Supplement: Additional file 1 — Schematic diagram of forming a PDDV. A cationic antigenic peptide containing 18 lysines (18K) and the antigenic epitope was designed and synthesized. An anionic plasmid containing the DNA sequence of the corresponding antigenic epitope sequence and mouse GM-CSF was constructed. The cationic peptides and corresponding anionic plasmids form virus-like particles through electrostatic interactions at an appropriate charge ratio of peptide and DNA. [file 1756-3305-3-109-S1.PDF]
